# Supplementary material for: EMS1 and BRI1 control separate biological processes via extracellular domain diversity and intracellular domain conservation
Source: Nat Commun. 2019 Sep 13;10:4165. doi: 10.1038/s41467-019-12112-w (PMC6744412; doi:10.1038/s41467-019-12112-w)
Supplement: Supplementary file 1 — Supplementary Information [file 41467_2019_12112_MOESM1_ESM.pdf]

Supplementary Information

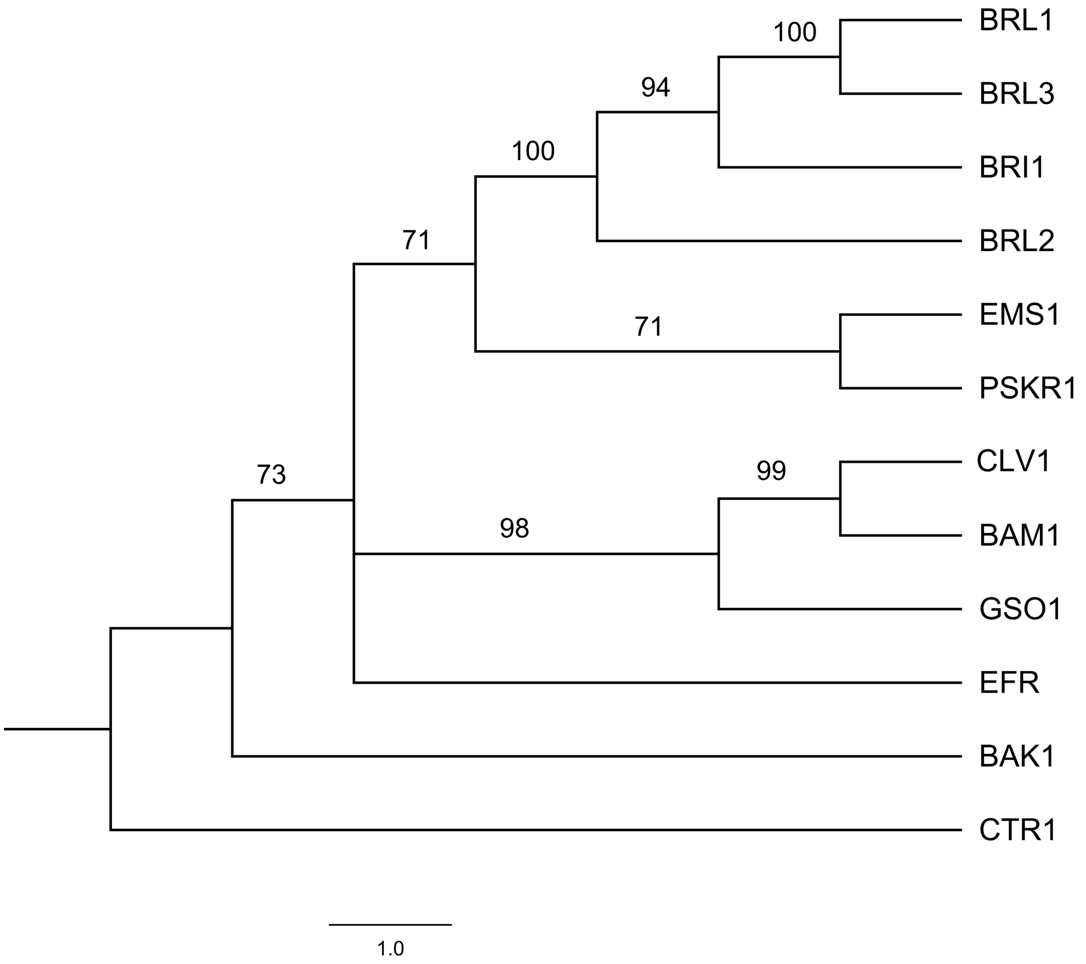

Supplementary Figure 1. The phylogenetic relationship of EMS1 and BRI1 homologues together with their closely related representatives of plant LRR-receptor like kinases (LRR-RLKs). The protein sequences were aligned with ClustalW. The phylogenetic tree was constructed using the Maximum Likelihood method with MEGAX software. Bootstrap values (in percentages) from 1000 replicates were shown next to the branches. Accessions numbers for the sequences used for alignments are as follows: BRI1 (AT4G39400), EMS1 (AT5G07280), BRL1 (AT1G55610), BRL3 (AT3G13380), BRL2 (AT1G14000), PSKR1 (AT2G02220), GSO1 (AT4G20140), CLV1 (AT1G75820), EFR (AT5G20480), BAM1 (AT5G65700), BAK1 (AT4G33430), CTR1 (AT5G03730).

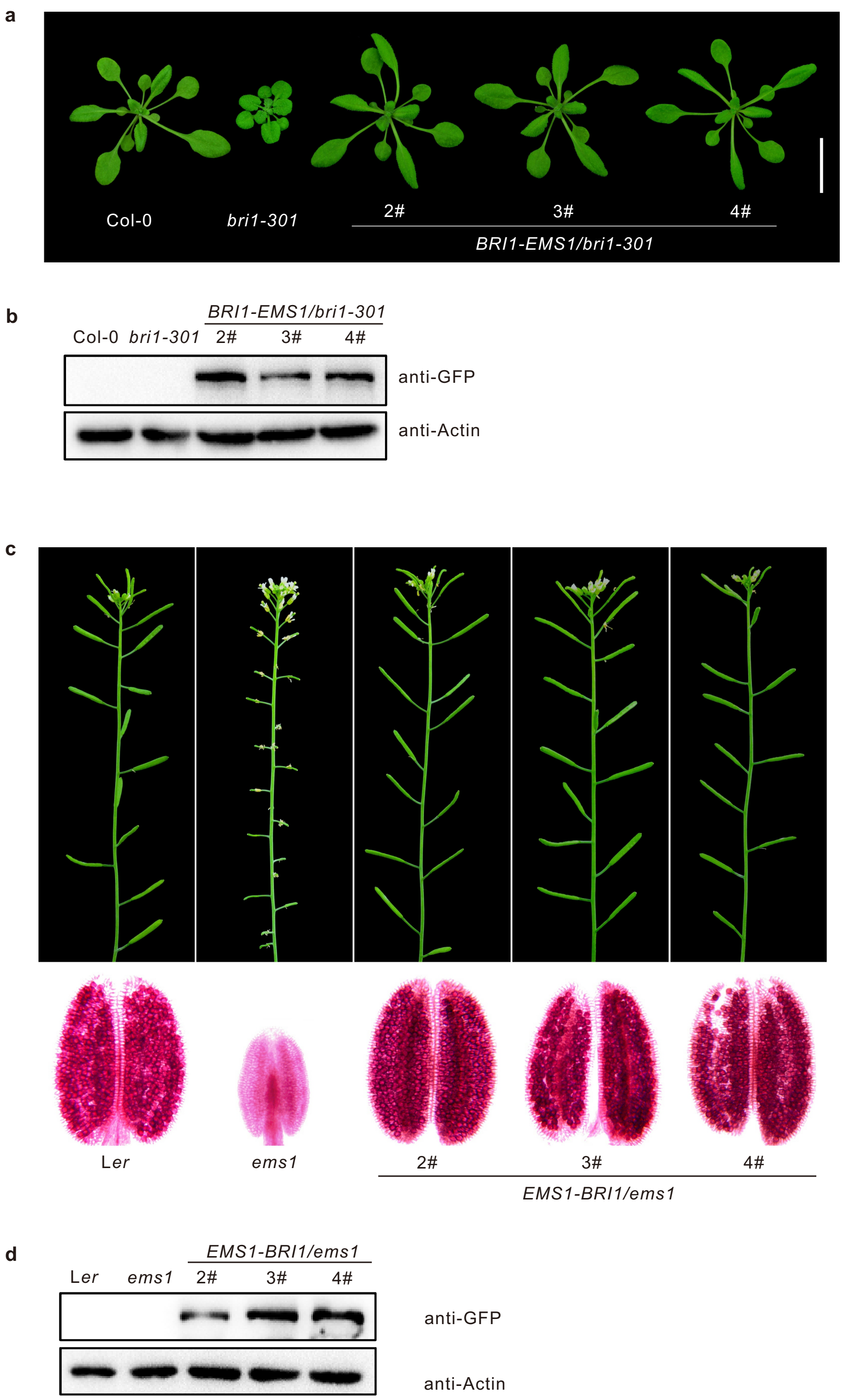

Supplementary Figure 2. Multiple independent transgenic lines expressing *BRI1-EMS1* and *EMS1-BRI1* rescue the phenotypes of *bri1-301* and *ems1*, respectively. (**a**) Phenotypes of 4-week-old transgenic lines expressing *BRI1-EMS1* under the *BRI1* promoter in *bri1-301* background. Scale bar, 2.0 cm. (**b**) Protein expression levels of the transgenes with GFP tag in the rosette leaves of the corresponding plants shown in (**a**) were detected with anti-GFP antibody. Actin served as the loading control. (**c**) Phenotypes of 6-week-old transgenic lines expressing *EMS1-BRI1* under the *EMS1* promoter in *ems1* background. Primary inflorescences (top) and Alexander staining of pollen grains in mature anthers (bottom) showing the fertility phenotypes of the transgenic plants. (**d**) Protein expression levels of the transgenes with GFP tag in the inflorescences of the corresponding plants shown in (**c**) were detected with anti-GFP antibody. Actin served as the loading control.

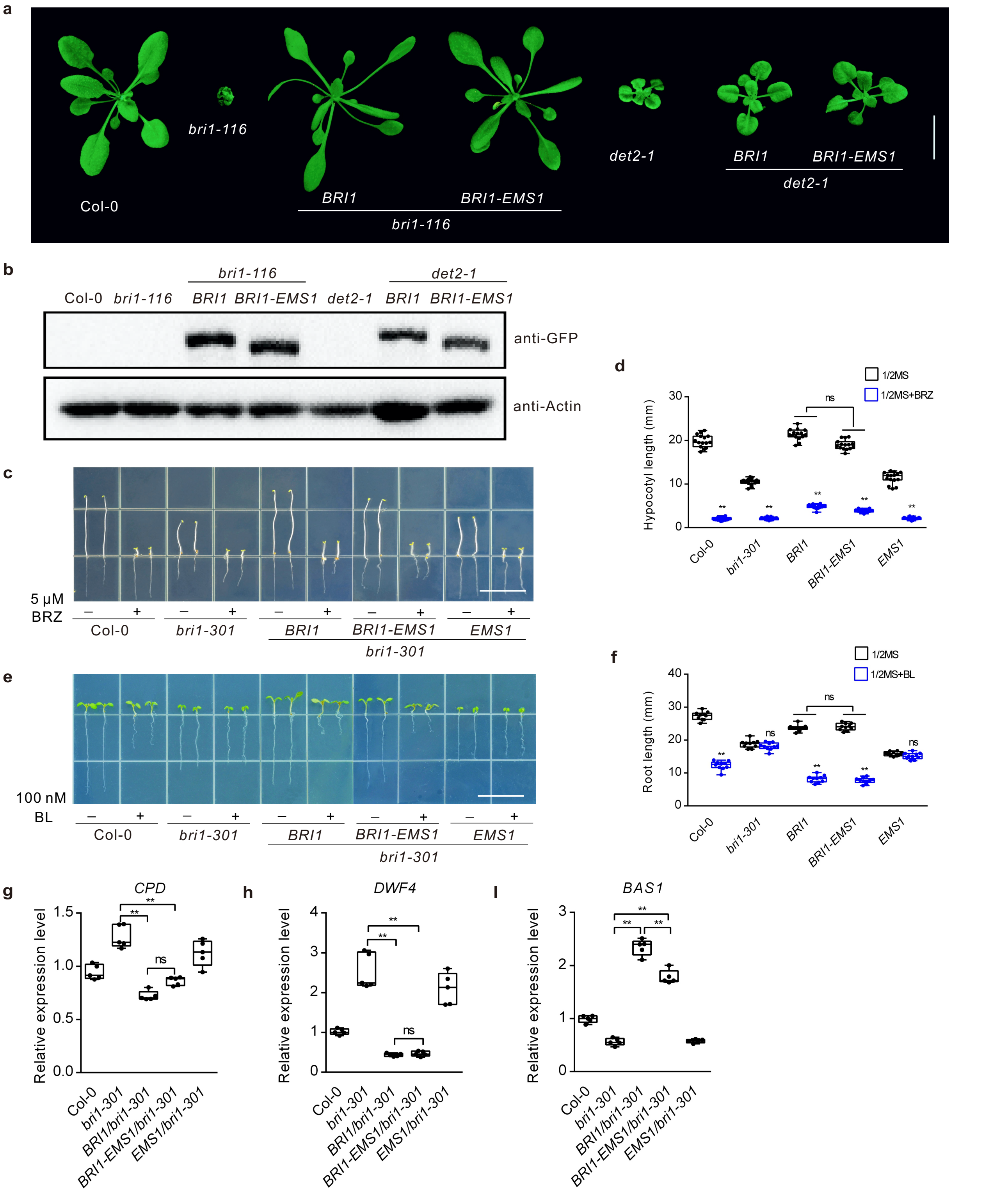

Supplementary Figure 3. Chimeric receptor kinase BRI1-EMS1 functions rescue *bri1-301* phenotypes. (**a**) Chimeric receptor kinase BRI1-EMS1 completely rescued the null mutant *bri1-116* and partially suppressed *det2-1* phenotypes. Scale bar, 2.0 cm. (**b**) Protein expression levels of the transgenes with GFP tag in the rosette leaves of the corresponding plants shown in (**a**) were detected with anti-GFP antibody. Actin serves as the loading control. (**c-d**) The transgenic lines of *BRI1-EMS1* and *BRI1* in *bri1-301* background had similar response to brassinazole (BRZ) or brassinolide (BL) treatment. 5-day-old dark-grown seedlings in 1/2 MS medium with or without 5 μM BRZ were shown in (**c**). Scale bar, 1.5 cm. Measurements of hypocotyl length were plotted as box plots displayed in (**c**), n = 15 seedlings. ***P* < 0.0001 as two-way ANOVA with a Sidak's multiple comparison test. (**e-h**) Root growth measurements were from 7-day-old seedlings grown on 1/2 MS medium with or without 100 nM BL were shown in (e). Scale bar, 1.5 cm. Measurements of root length were plotted as box plots and displayed in (**f**), n = 10 seedlings. ***P* < 0.0001 as two-way ANOVA with a Sidak's multiple comparison test. (**g-i**) Similar response was found in BR regulated genes in the transgenic lines of BRI1-EMS1 and BRI1. Quantitative real-time PCR analysis of BR biosynthetic genes *CPD* and *DWF4* or BR inactivation gene *BAS1* in 4-week-old plants. n = 5 biological replicates. ***P* < 0.0001 (one-way ANOVA with Tukey's test).

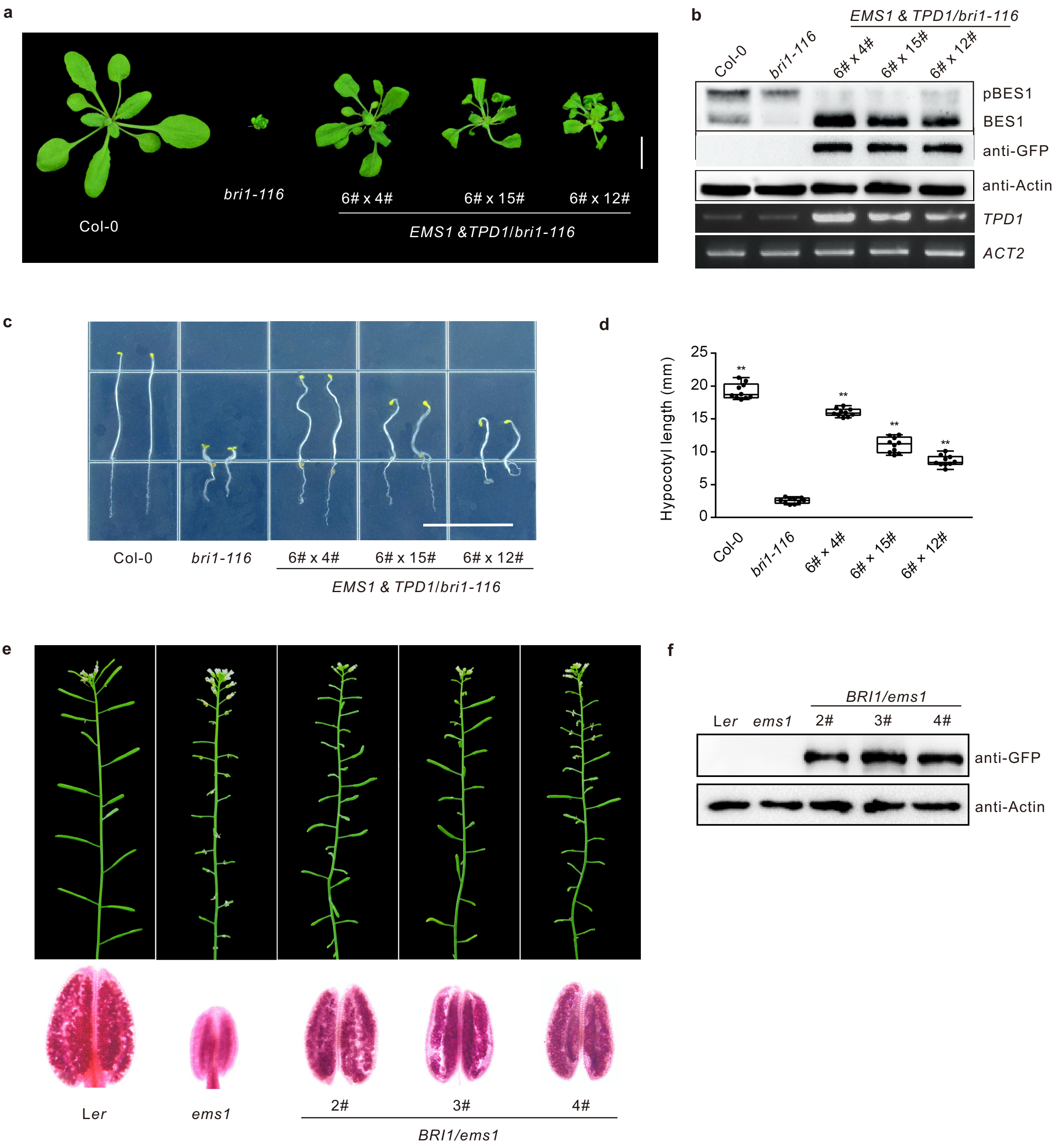

Supplementary Figure 4. Multiple independent transgenic lines co-expressing *EMS1* & *TPD1* and *BRI1* rescue the phenotypes of *bri1-116* and *ems1*, respectively. (**a**) Phenotypes of 4-week-old plants co-expressing *EMS1* & *TPD1* under the *BRI1* promoter in *bri1-116* background. Scale bar, 2.0 cm. (**b**) Analyses of the expression levels of the transgenes in the 10-day-old seedlings of the in corresponding plants shown in (**a**). Phosphorylated BES1 (pBES1) and dephosphorylated BES1 were detected with BES1 antibodies. Proteins with GFP tag were detected with anti-GFP antibody. Actin serves as the loading control. *TPD1* expression levels were detected using RT-PCR. *ACT2* served as an internal control. (**c-d**) Hypocotyl elongation of 5-day-old dark-grown transgenic seedlings was shown in (**c**). Scale bar, 1.5 cm. Measurements of rosette width were plotted as box plots and displayed in (**d**), n = 10 seedlings. ** $P < 0.0001$ as one-way ANOVA with Tukey's test. (**e**) Phenotypes of 6-week-old transgenic lines expressed *BRI1* under the *EMS1* promoter in *ems1* background. Primary inflorescences (top) and Alexander staining of pollen grains in mature anthers (bottom) showing the fertility phenotypes of transgenic plants. (**f**) Protein expression levels of the transgenes with GFP tag in the inflorescences of the corresponding plants shown in (**e**) were detected with anti-GFP antibody. Actin serves as the loading control.

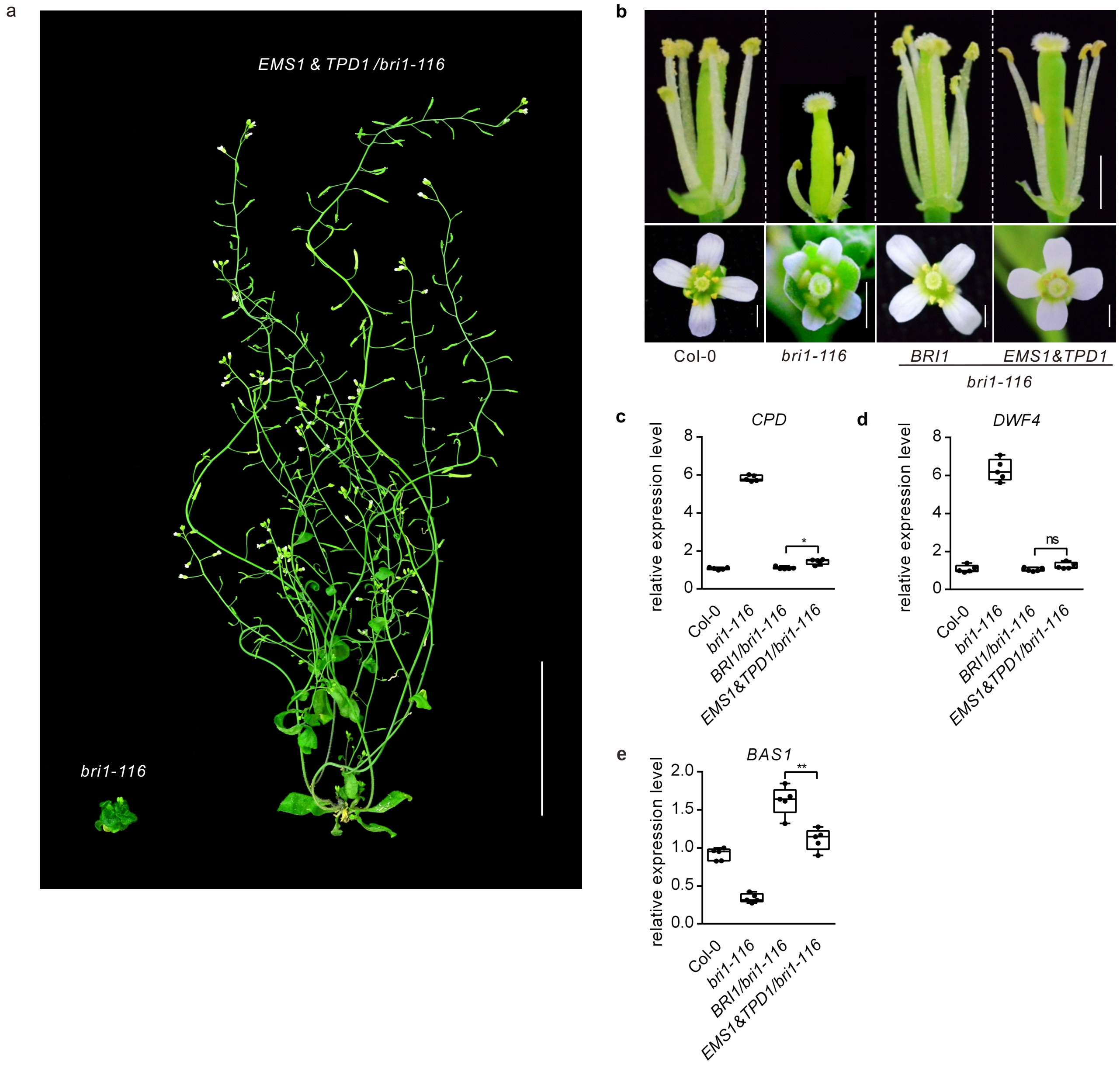

Supplementary Figure 5. Co-expressing *EMS1* & *TPD1* rescues the null *bri1-116* phenotypes independent of BRs. (**a**) Phenotypes of 7-week-old plants that co-expressing *EMS1* & *TPD1* under *BRI1* promoter in null *bri1-116* mutants. Scale bar, 5 cm. (**b**) Mature flowers of Col-0, *bri1-116*, expression of *BRI1* and co-expression of *EMS1* & *TPD1* in *bri1-116*. Showing co-expressing *EMS1* & *TPD1* in *bri1-116* with normal stamen filaments and pollen grains. Scale bars, 1 mm. (**c**-**e**) Quantitative real-time PCR analysis of BR biosynthetic genes *CPD* and *DWF*4 or BR inactivation gene *BAS1* in 4-week-old plants. n = 5 biological replicates. ***P* < 0.0001 (one-way ANOVA with Tukey's test). *EMS1* & *TPD1*/*bri1-116* (6# X 15#) was analyzed in (**a**-**e**).

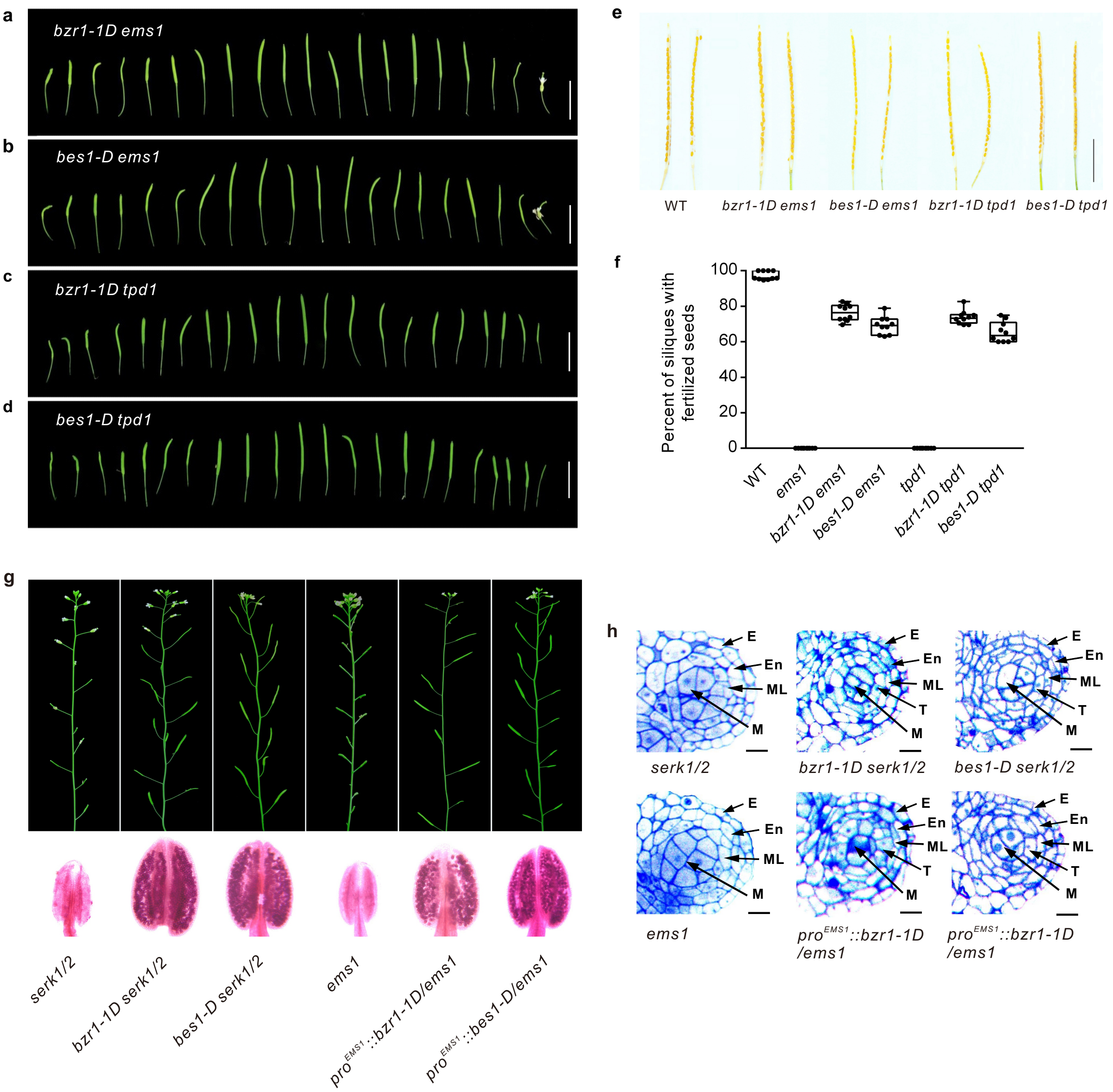

Supplementary Figure 6. Dominant mutants *bzr1-1D* and *bes1-D* of BRI1-BRs signaling rescue EMS1 signaling mutants. (**a-d**) All siliques in a branch of 6-week-old plants of *bzr1-1Dems1*, *bes1-Dems1*, *bzr1-1Dtpd1* and *bes1-Dtpd1*, respectively. Scale bars, 1 cm. (**e**) Seeds in mature siliques of WT(Col-0), *bzr1-1Dems1*, *bes1-Dems1*, *bzr1-1Dtpd1* and *bes1-Dtpd1*, Scale bar, 5 mm. (**f**) Percent of siliques with fertilized seeds on the primary inflorescences of WT(Col-0), *bzr1-1Dems1*, *bes1-Dems1*, *bzr1-1Dtpd1* and *bes1-Dtpd1.* n = 10. (**g**) Primary inflorescences of triple mutants *bzr1-1Dserk1/2* and *bes1-Dserk1/2* and transgenic plants of *pro*^{EMS1}*::bzr1-1D* and *pro*^{EMS1}*::bes1-D* in *ems1* background. These triple mutants and transgenic plants were fertile as indicated by the long siliques with seeds, whereas *serk1/2* double mutant or *ems1* mutant were sterile as indicated by short siliques without seeds. Alexander staining of pollen grains in mature anthers showing viable pollen grains in the triple mutants and transgenic plants, but not in the *serk1/2* double and *ems1* single mutant plants. (**h**) Semi-thin sections of stage-5 anthers showing anther cell layers in *serk1/2*, *bzr1-1Dserk1/2*, *bes1-Dserk1/2*, *ems1*, *pro*^{EMS1}*::bzr1-1D* in *ems1* and *pro*^{EMS1}*::bes1-D* in *ems1* background. E, epidermis; En, endothecium; ML, the middle layer; T, tapetal cells; and M, microsporocytes. Scale bars, 10 µm.

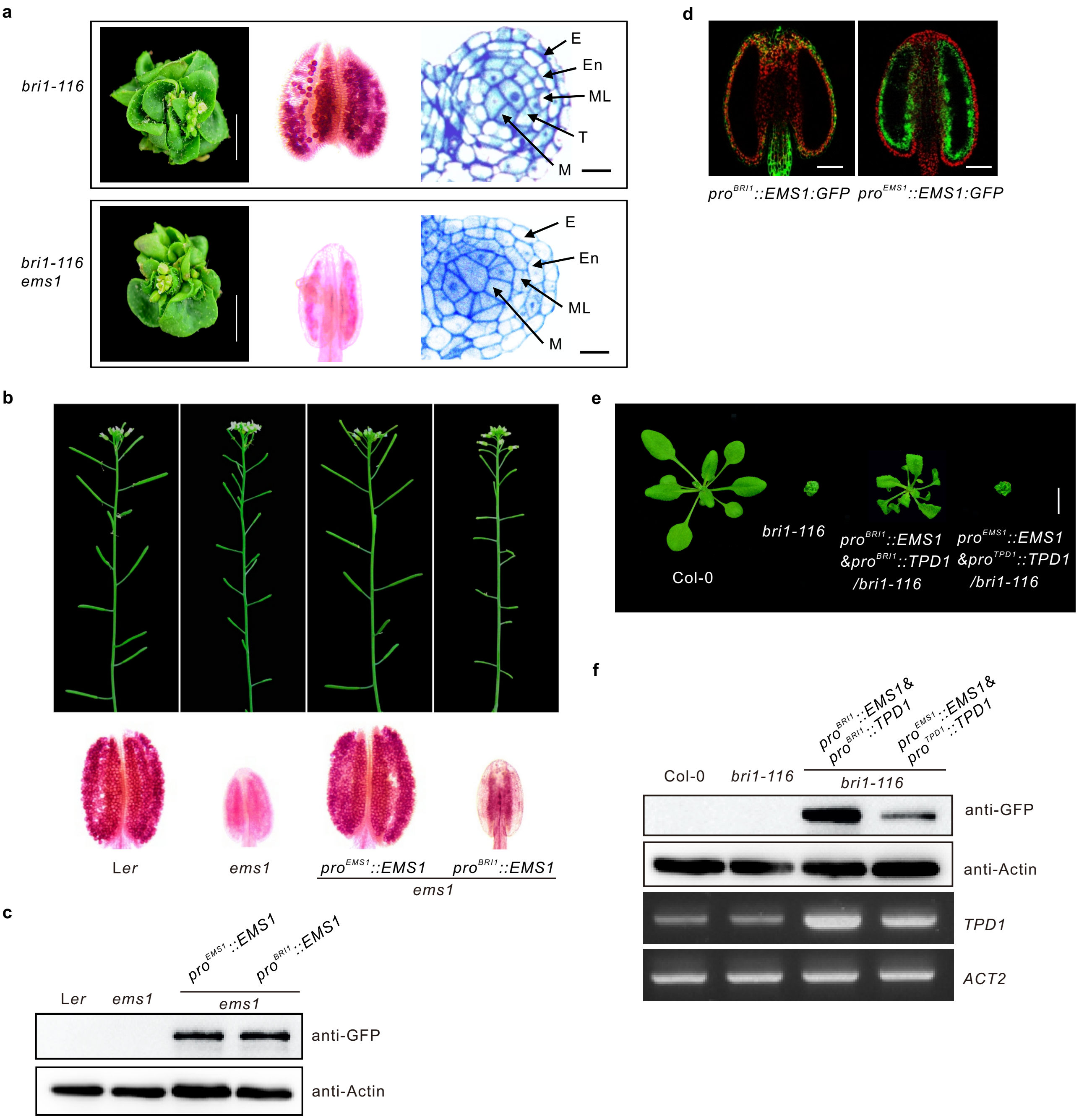

Supplementary Figure 7. EMS1 specifies tapeta in absence of *BRI1* expression. (**a**) Phenotypes of 7-week-old double *bri1-116ems1* and single *bri1-116* mutants shown on the left. Scale bars, 5 mm. Alexander staining of pollen in mature anthers showing viable pollen grains in *bri1-116* mutants, but inviable pollen grains in *bri1-116ems1* double mutants that had additive phenotypes of both single mutants. Semi-thin sections of stage-5 anther lobes showing normal anther cell differentiation with tapetal cells in *bri1-116* mutants, but lack of tapetal cells in *bri1-116ems1* mutants. Scale bars, 10 μm. (**b**) Primary inflorescences (top) and Alexander staining of pollen grains in mature anthers (bottom) showing phenotypes of transgenic lines expressing *EMS1* under *EMS1* and *BRI1* promoter in *ems1* background, respectively. (**c**) Protein expression levels of the transgenes with GFP tag in the inflorescences of the corresponding plants shown in (**b**) were detected with anti-GFP antibody. Actin served as the loading control. (**d**) Confocal images reveal the significant amount of natural EMS1 and insignificant amount of natural BRI1 in tapeta. The expression of *EMS1:GFP* in an anther from the transgenic plants of *pro*^{BRI1}*::EMS1:GFP* and in an anther from the transgenic plants of *pro*^{EMS1}*::EMS1:GFP* was indicated by GFP fluorescence. Green, GFP signal; Red, auto-fluorescence. Scale bars, 50 μm. (**e**) Phenotypes of 4-week-old transgenic lines co-expressing *EMS1* and *TPD1* under *BRI1* promoter or their native promoters in *bri1-116* background. Scale bar, 1 cm. (**f**) Analyses of the expression levels of the transgenes in the rosette leaves of the corresponding plants shown in (**e**). Proteins with GFP tag were detected with anti-GFP antibody. Actin served the loading control. *TPD1* expression levels were detected by semi-quantitative RT-PCR. *ACT2* serves as an internal control.

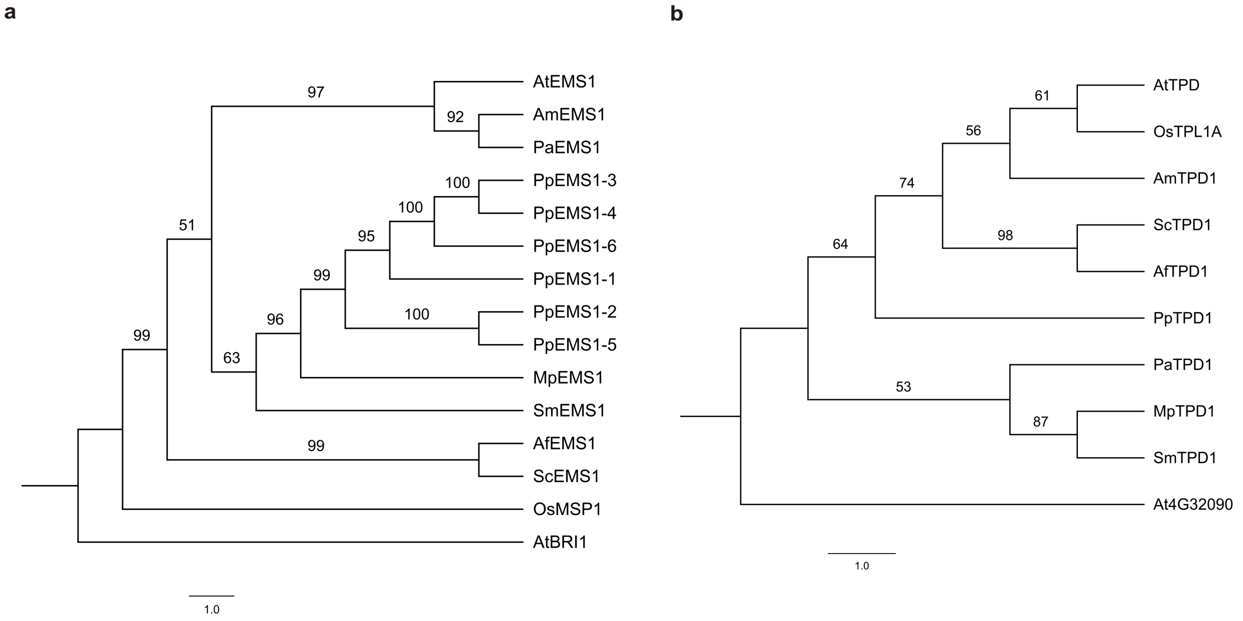

Supplementary Figure 8. The phylogenetic tree of EMS1 and TPD1. (**a**-**b**) the protein sequences of EMS1 and TPD1 from *Arabidopsis thaliana* (At), *Oryza sativa* (Os), *Amborella trichopoda* (Am), *Picea abies* (Pa), *Azolla filiculoides* (Af), *Salvinia cucullata* (Sc), *Selaginella moellendorffii* (Sm), *Physcomitrella patens* (Pp), *Marchantia polymorpha* (Mp). The protein sequences were aligned with ClustalW, respectively. The phylogenetic tree was constructed using the Maximum Likelihood method with MEGAX software. Bootstrap values (in percentages) from 1000 replicates were shown next to the branches. Accessions numbers for the alignments are as follows: AtBRI1 (AT4G39400), AtEMS1 (AT5G07280), OsMSP1 (LOC_Os01g68870.1), AmEMS1 (evm_27.model.AmTr_v1.0_scaffold00009.24), PaEMS1 (MA_1913g0010), AfEMS1 (Azfi_s0017.g014644), ScEMS1 (Sacu_v1.1_s0054.g014268), SmEMS1 (Sm99902), PpEMS1-1 (Pp3c1_41620V3.1), PpEMS1-2 (Pp3c14_16840V3.1), PpEMS1-3 (Pp3c22_12040V3.1), PpEMS1-4 (Pp3c19_18410V3.2), PpEMS1-5 (Pp3c17_21540V3.3), PpEMS1-6 (Pp3c1_16110V3.4), MpEMS1 (Mapoly0011s0213.1); AtTPD1 (AT4G24972), OsTPL1A (LOC_Os12g28750.1), AmTPD1 (evm_27.model.AmTr_v1.0_scaffold00047.41), PaTPD1 (MA_10427288g0010), AfTPD1 (Azfi_s0003.g008001), ScTPD1 (Sacu_v1.1_s0032.g010785), SmTPD1 (Sm113463), PpTPD1 (Pp3c22_22420V3.1), MpTPD1 (Mapoly0020s0056.1).

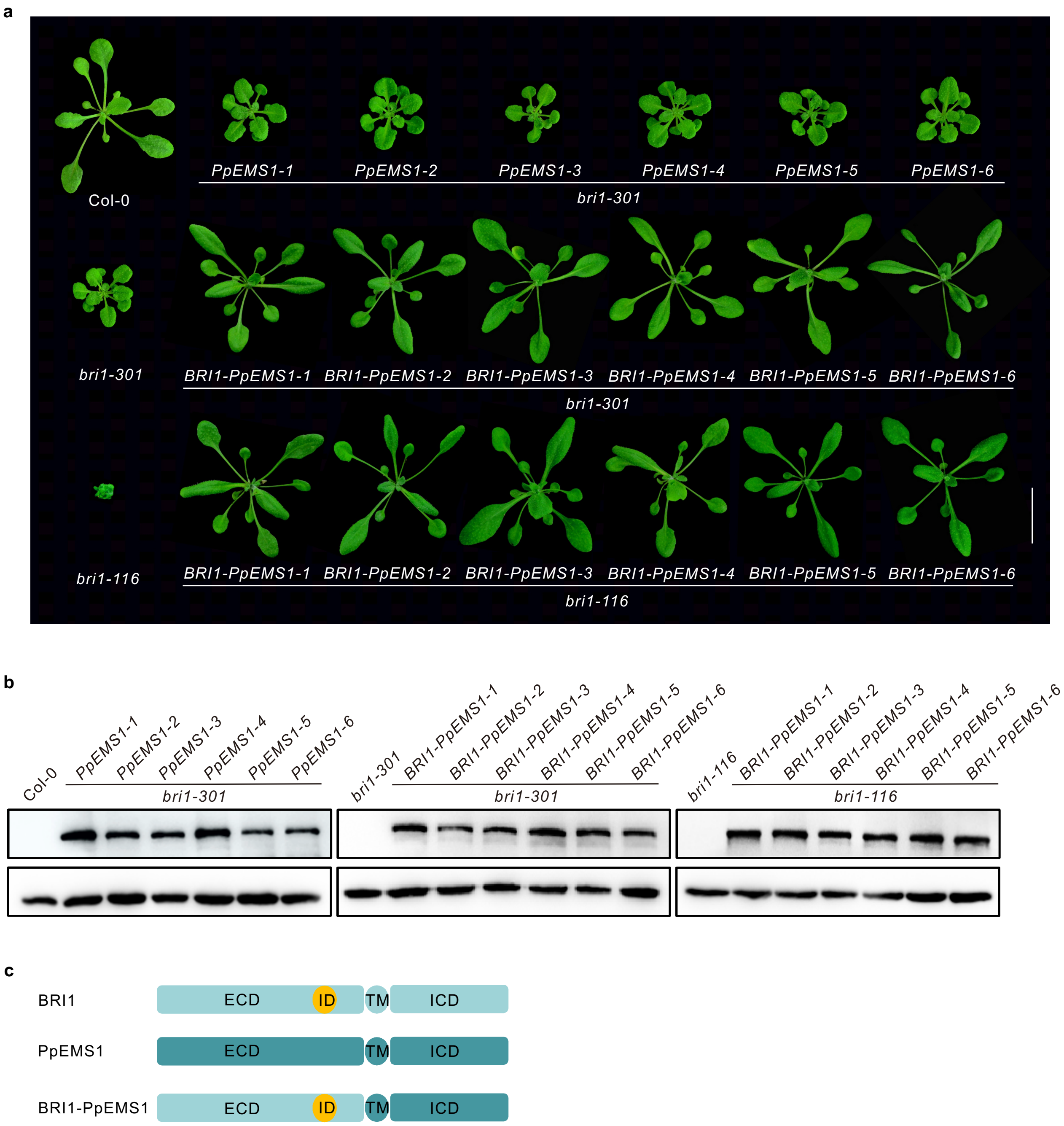

Supplementary Figure 9. The intracellular domains of PpEMS1 can replace the intracellular domain of BRI1 . (**a**) Phenotypes of 4-week-old Col-0, *bri1-301*, *bri1-116* and transgenic lines expressing all six *EMS1* homologues isolated from *P. patens* (*PpEMS1-1* to *PpEMS1-6*) in *bri1-301* mutants and their chimeric receptor kinases with the extracellular domain of BRI1 (*BRI1-PpEMS1-1* to *BRI1-PpEMS1-6*) in *bri1-301* and *bri1-116* mutants, respectively. Scale bar, 2 cm. (**b**) Protein expression levels of the transgenes with GFP-tag in corresponding plants shown in (**a**) were detected using the rosette leaves of plants with anti-GFP antibody. Actin served as the loading control. (**c**) A schematic diagram of receptor kinases BRI1 and PpEMS1 together with their chimeric receptor kinases. The domains of BRI1 were labeled in light blue while the domains of PpEMS1 were labeled in dark blue.

Supplementary Table 1. Primers used in this study.

Primer name	Purpose	Sequence (5'to3')
BRI1-F-KpnI	Cloning	GCGGTACCATGAAGACTTTTTCAAGCTTCTTTC
BRI1-R-SalI	Cloning	GCGGTCGACTAATTTTCCTTCAGGAACTTCTT
EMS1-F-KpnI	Cloning	GGTACCATGGCGTTTCTTACCGCATTGTTC
EMS1-R-SalI	Cloning	GTCGACTCCTATCTCCTTAAGAGCCTTCAAC
BRI1-EMS1-F	Overlapping	GAAGGAGACCAGCGTGGGGGATTGCAG
BRI1-EMS1-R	Overlapping	CTGCAATCCCCCACGCTGGTCTCCTTC
BRI1-PSKR-F	Overlapping	CGTCCCTTGCTGGTGACATTGGAATGG
BRI1-PSKR-R	Overlapping	CCATTCCAATGTCACCAGCAAGGGACG
PSKR1-R-SalI	Cloning	GTCGACGACATCATCAAGCCAAGAGAC
BRI1-GSO1-F	Overlapping	CCAGCGTCCCTTGCCAGATCAGTTG
BRI1-GSO1 -R	Overlapping	CAACTGATCTGGCAAGGGACGCTGG
GSO1-R-SalI	Cloning	GTCGACCAGCTTCTTATAACCGGCCGTTC
BRI1-CLV1-F	Overlapping	GACCAGCGTCCCTTGTAATCACGGTTATC
BRI1-CLV1-R	Overlapping	GATAACCGTGATTACAAGGGACGCTGGTC
CLV1-R-Sal1	Cloning	GTCGACGAACGCGATCAAGTTCGCCACG
BRI1-BAM1- F	Overlapping	GACCAGCGTCCCTTAAGTTATTGCTTGTTC
BRI1-BAM1- R	Overlapping	GAACAAGCAATAACTTAAGGGACGCTGGTC
BAM1 -R- SalI	Cloning	GTCGACTAGATTGAGTAGATCCGGCGGAC
BRI1-EFR-F	Overlapping	GACCAGCGTCCCTTAGAAAGAAAGTTGTC
BRI1-EFR-R	Overlapping	GACAACTTTCTTTCTAAGGGACGCTGGTC
EFR-R- SalI	Cloning	GCGTCGACCATAGTATGCATGTCCGTAT
BRI1-BAK1-F	Overlapping	CGTCCCTTGCTGGTGGAGTTGCTG
BRI1-BAK1-R	Overlapping	CAGCAACTCCACCAGCAAGGGACG
BAK1-R-SalI	Cloning	GTCGACTCTTGGACCCGAGGGGTATTC
EMS1-PSKR1-F	Overlapping	CGTTTAGGAGATCTTTTGGACTCAAC
EMS1-PSKR1-R	Overlapping	GTTGAGTCCAAAAGATCTCCTAAACG
EMS1-BRI1-F	Overlapping	GTTGAGGAGTGCTTCCCTTGCTGGTAG
EMS1-BRI1-R	Overlapping	CTACCAGCAAGGGAAGCACTCCTCAAC
PpEMS1-1-F-SmaI	Cloning	CCCGGGATGGATAGGCCAAATCAAGC
PpEMS1-1-R-SalI	Cloning	CGTCGACCTGAGCATCGTCGTGTGTAG
PpEMS1-2-F-KpnI	Cloning	GGTACCATGCTCGGGTCGCCGCG
PpEMS1-2-R-SalI	Cloning	CGTCGACTACGTGGTCCTGGTCCTCG
PpEMS1-3-F-SmaI	Cloning	CCCGGGATGAATATCTCCAATGTAGTACAG
PpEMS1-3-R-SmaI	Cloning	CCCGGGATACAGGGGCAAGTGCCGTG
PpEMS1-4-F-KpnI	Cloning	GGTACCATGCAACTGAGGTTGCTCATC
PpEMS1-4-R-SalI	Cloning	GTCGACCACATTCGTCAGAGTTTTGAAC
PpEMS1-5-F-SmaI	Cloning	CCCGGGATGCTCGGTTCGTTGCAAATTC
PpEMS1-5-R-SalI	Cloning	GTCGACTGCGCGATCCTGGTCTTCAAT
PpEMS1-6-F-SmaI	Cloning	CCCGGGATGAGTATCTTCACTCTCGCACTG
PpEMS1-6-R-SalI	Cloning	GTCGACCAAATTGCTCGAGGTCGAAAAC
BRI1-PpEMS1-1-F	Overlapping	CTGGTGTGAAAGAACCTCTGAGCATCAATG
BRI1-PpEMS1-1-R	Overlapping	CATTGATGCTCAGAGGTTCTTTCACACCAG

BRI1-PpEMS1-3-F	Overlapping	CGTCCCTTGCTGGTGCTCTTCTGGG
BRI1-PpEMS1-3-R	Overlapping	CCCAGAAGAGCACCAGCAAGGGACG
BRI1-PpEMS1-4-F	Overlapping	GTCCCTTGCTGGTGCGCTGTTGGG
BRI1-PpEMS1-4-R	Overlapping	CCCAACAGCGCACCAGCAAGGGAC
BRI1-PpEMS1-5-F	Overlapping	GTCCCTTGCTGGTGCCATTCTGGGGATC
BRI1-PpEMS1-5-R	Overlapping	GATCCCCAGAATGGCACCAGCAAGGGAC
BRI1-PpEMS1-6-F	Overlapping	GTCCCTTGCTGGTACGGTCATGGGTATC
BRI1-PpEMS1-6-R	Overlapping	GATACCCATGACCGTACCAGCAAGGGAC
AtTPD-F-KpnI	Cloning, RT	GGTACCATGAACCGACGGCGACTTTTG
AtTPD-R-SalI	Cloning, RT	GTCGACCTAAGCACATGTCACGAAGGC
PpTPD1-F-KpnI	Cloning, RT	GGTACCATGAGTGCCATGATCCTTGTC
PpTPD1-R-SalI	Cloning, RT	GTCGACTCATGGCCATTTACACGTGAC
BES1-F-KpnI	Cloning	GGTACCATGAAAAGATTCTTCTATAATTC
BES1-R-SmaI	Cloning	CCCGGGTCAACTATGAGCTTTACCATTTC
BZR1-F-KpnI	Cloning	GGTACCATGACTTCGGATGGAGCTACGTC
BZR1-R-BamHI	Cloning	GGATCCTCAACCACGAGCCTTCCCATTTC
ProAtBRI1-F-EcoRI	Cloning	GAATTCTAACATCAATGGCTAAG
ProAtBRI1-R-KpnI	Cloning	GGTACCCTTCTCAAGAGTTTGTGAGAGAG
ProAtEMS1-F-SacI	Cloning	GAGCTCCAGAGAGAACCAATGCAAC
ProAtEMS1-R-KpnI	Cloning	GGTACCGTTCTTTTAGAGAAGGAGG
ProAtTPD1-F-SacI	Cloning	GAGCTCACATAGAGCTTGCATATATTTGG
ProAtTPD1-R-KpnI	Cloning	GGTACCGTGCGTAGACGTCGAAGAAC
AtACT2-qRT-F	qRT	ACTCTCCCGCTATGTATGTCG
AtACT2-qRT-R	qRT	AGAAACCCTCGTAGATTGGC
AtACT2-RT-R	RT	TGGACCTGCCTCATCATACTC
AtCPD-qRT-F	qRT	GCAATGACGGATGTTGAGAT
AtCPD-qRT-R	qRT	CAAGGGTTGAAAGTGCGAGC
AtDWF4-qRT-F	qRT	AACAGACGATGATCTTTTGGG
AtDWF4-qRT-R	qRT	CTTCAACGGCTTTAGGGCAA
AtBAS1-qRT-F	qRT	GCCAAATTGACACTCGCTGTAA
AtBAS1-qRT-R	qRT	GACGGTAGGTGCATGCTGATAA
Ds-LB	genotyping	CGTTCCGTTTTCGTTTTTTACC
ems1-gt-F	genotyping	AACAAACCCCGTCAGCTTTA
ems1-gt-R	genotyping	ACCGGAGAAGTGGTTGTCAC
EMS1-F1-LP-1950	genotyping	GCCCATTCCCTTCAGTAAAC
EMS1-R1-RP-213	genotyping	GGAGAGCGAGTTGACTCGTC
tpd1-gt-F	genotyping	CTTTTGGTATCGGCGACACTG
tpd1-gt-R	genotyping	CTAAGCACATGTCACGAAGGC
serk1-1-LP	genotyping	ATACACAAAAGTGAAACGGCG
serk1-1-RP	genotyping	TTAGACGAAGAATTCGAAGCG
serk2-1-LP	genotyping	AGTGAAGAGCGAGAAGGAACC
serk2-1-RP	genotyping	AAGGCTTAGGCTTTTGTTTGG
LBb1.3	genotyping	ATTTTGCCGATTTCGGAAC
bri1-116-F	genotyping	TGGCGAGTTACCGATGGATACG

bri1-116-R	genotyping	CTCTTAGATCACCTACCTCATCAGG
bri1-116-T-R	genotyping	GACCCAAGGAAAATCGGACTGACC

Supplementary Table 2. Constructs generated in this study.

**Construct**	**Construct backbone**	**Purpose**
pro^{BRI1}*::CHF3-GFP*	pCHF3-GFP	Construct backbone
pro^{BRI1}*:: 1300-GFP*	pCAMBIA1300-GFP	Construct backbone
pro^{BRI1}*:: 1300*	pCAMBIA1300	Construct backbone
pro^{EMS1}*::1300-GFP*	pCAMBIA1300-GFP	Construct backbone
pro^{EMS1}*::CHF3-GFP*	pCHF3-GFP	Construct backbone
pro^{TPD1}*::1300*	pCAMBIA1300	Construct backbone
pro^{BRI1}*:: BRI1:GFP*	pro^{BRI1}::CHF3-GFP	Complementary assay
pro^{BRI1}*:: EMS1:GFP-1*	pro^{BRI1}::CHF3-GFP	Complementary assay
pro^{BRI1}*:: BRI1-EMS1:GFP*	pro^{BRI1}::CHF3-GFP	Complementary assay
pro^{BRI1}*::BRI1-PSKR1:GFP*	pro^{BRI1}::CHF3-GFP	Complementary assay
pro^{BRI1}*::BRI1-GSO1:GFP*	pro^{BRI1}::CHF3-GFP	Complementary assay
pro^{BRI1}*::BRI1-CLV1:GFP*	pro^{BRI1}::CHF3-GFP	Complementary assay
pro^{BRI1}*::BRI1-BAM1:GFP*	pro^{BRI1}::CHF3-GFP	Complementary assay
pro^{BRI1}*::BRI1-EFR:GFP*	pro^{BRI1}::CHF3-GFP	Complementary assay
pro^{BRI1}*::BRI1-BAK1:GFP*	pro^{BRI1}::CHF3-GFP	Complementary assay
pro^{BRI1}*::TPD1*	pro^{BRI1}::1300	Complementary assay
pro^{TPD1}*::TPD1*	pro^{TPD1}::1300	Complementary assay
pro^{EMS1}*::EMS1:GFP-1*	pro^{EMS1}::1300-GFP	Complementary assay
pro^{EMS1}*::EMS1:GFP-2*	pro^{EMS1}::CHF3-GFP	Complementary assay
pro^{EMS1}*::EMS1-BRI1:GFP*	pro^{EMS1}::1300-GFP	Complementary assay
pro^{EMS1}*::EMS1-PSKR1:GFP*	pro^{EMS1}::1300-GFP	Complementary assay
pro^{EMS1}*::PpEMS1-1:GFP*	pro^{EMS1}::1300-GFP	Complementary assay
pro^{EMS1}*::PpEMS1-2:GFP*	pro^{EMS1}::1300-GFP	Complementary assay
pro^{BRI1}*::PpEMS1-1:GFP*	pro^{BRI1}::CHF3-GFP	Complementary assay
pro ^{BRI1}*::PpEMS1-2:GFP*	pro^{BRI1}::CHF3-GFP	Complementary assay
pro^{BRI1}*::PpEMS1-3:GFP*	pro^{BRI1}::CHF3-GFP	Complementary assay
pro^{BRI1}*::PpEMS1-4:GFP*	pro^{BRI1}::CHF3-GFP	Complementary assay
pro^{BRI1}*::PpEMS1-5:GFP*	pro^{BRI1}::CHF3-GFP	Complementary assay
pro^{BRI1}*::PpEMS1-6:GFP*	pro^{BRI1}::CHF3-GFP	Complementary assay
pro^{BRI1}*::PpTPD1*	pro^{BRI1}::1300	Complementary assay
pro^{TPD1}*::PpTPD1*	pro^{TPD1}::1300	Complementary assay
pro^{BRI1}*::BRI1-PpEMS1-1:GFP*	pro^{BRI1}::CHF3-GFP	Complementary assay
pro^{BRI1}*::BRI1-PpEMS1-2:GFP*	pro^{BRI1}::CHF3-GFP	Complementary assay
pro^{BRI1}*::BRI1-PpEMS1-3:GFP*	pro^{BRI1}::CHF3-GFP	Complementary assay
pro^{BRI1}*::BRI1-PpEMS1-4:GFP*	pro^{BRI1}::CHF3-GFP	Complementary assay
pro^{BRI1}*::RI1-PpEMS1-5:GFP*	pro^{BRI1}::CHF3-GFP	Complementary assay
pro^{BRI1}*::BRI1-PpEMS1-6:GFP*	pro^{BRI1}::CHF3-GFP	Complementary assay
pro^{EMS1}*::bes1-D*	pro^{EMS1}::1300	Complementary assay
pro^{EMS1}*::bzr1-1D*	pro^{EMS1}::1300	Complementary assay
