# Supplementary material for: EMS1 and BRI1 control separate biological processes via extracellular domain diversity and intracellular domain conservation
Source: Nat Commun. 2019 Sep 13;10:4165. doi: 10.1038/s41467-019-12112-w (PMC6744412; doi:10.1038/s41467-019-12112-w)
Supplement: Supplementary file 3 — Reporting Summary [file 41467_2019_12112_MOESM3_ESM.pdf]

nature research

Corresponding author(s): Guang Wu and Xiaoping She

Last updated by author(s): 2019-8-20

Reporting Summary

Nature Research wishes to improve the reproducibility of the work that we publish. This form provides structure for consistency and transparency in reporting. For further information on Nature Research policies, see Authors & Referees and the Editorial Policy Checklist .

Statistics

For all statistical analyses, confirm that the following items are present in the figure legend, table legend, main text, or Methods section.

n/a	Confirmed	
☐	☒	The exact sample size (*n*) for each experimental group/condition, given as a discrete number and unit of measurement
☐	☒	A statement on whether measurements were taken from distinct samples or whether the same sample was measured repeatedly
☐	☒	The statistical test(s) used AND whether they are one- or two-sided<br>*Only common tests should be described solely by name; describe more complex techniques in the Methods section.*
☒	☐	A description of all covariates tested
☐	☒	A description of any assumptions or corrections, such as tests of normality and adjustment for multiple comparisons
☐	☒	A full description of the statistical parameters including central tendency (e.g. means) or other basic estimates (e.g. regression coefficient) AND variation (e.g. standard deviation) or associated estimates of uncertainty (e.g. confidence intervals)
☐	☒	For null hypothesis testing, the test statistic (e.g. *F*, *t*, *r*) with confidence intervals, effect sizes, degrees of freedom and *P* value noted<br>*Give P values as exact values whenever suitable.*
☒	☐	For Bayesian analysis, information on the choice of priors and Markov chain Monte Carlo settings
☒	☐	For hierarchical and complex designs, identification of the appropriate level for tests and full reporting of outcomes
☒	☐	Estimates of effect sizes (e.g. Cohen's *d*, Pearson's *r*), indicating how they were calculated

Our web collection on statistics for biologists contains articles on many of the points above.

Software and code

Policy information about availability of computer code

Data collection	N/A
Data analysis	Alignments: MEGAX<br>phylogenetic tree construct: MEGAX and FigTree v1.4.3.<br>Statistical analysis GraphPad Prism 6.<br>Image analysis: ImageJ 1.50i

For manuscripts utilizing custom algorithms or software that are central to the research but not yet described in published literature, software must be made available to editors/reviewers. We strongly encourage code deposition in a community repository (e.g. GitHub). See the Nature Research guidelines for submitting code & software for further information.

Data

Policy information about availability of data

All manuscripts must include a data availability statement. This statement should provide the following information, where applicable:

- Accession codes, unique identifiers, or web links for publicly available datasets
- A list of figures that have associated raw data
- A description of any restrictions on data availability

The data supporting the findings of this study are available within the paper and its Supplementary Information files. Source Data for Figs. 1-4 and Supplementary Figs. 2-7, 9 are provided with the paper.

Field-specific reporting

Please select the one below that is the best fit for your research. If you are not sure, read the appropriate sections before making your selection.

- [x] Life sciences
- [ ] Behavioural & social sciences
- [ ] Ecological, evolutionary & environmental sciences

For a reference copy of the document with all sections, see nature.com/documents/nr-reporting-summary-flat.pdf

Life sciences study design

All studies must disclose on these points even when the disclosure is negative.

Sample size	For root length, rosette width and hypocotyl length analysis, the sample size was used according to publications with n ≥ 10. Given the sample size, adequate statistical analysis was performed.
Data exclusions	No data excluded.
Replication	All attempts at replication were successful.
Randomization	All samples were allocated randomly into experimental groups.
Blinding	All experiments were blinded during data acquisitions and analyses.

Reporting for specific materials, systems and methods

We require information from authors about some types of materials, experimental systems and methods used in many studies. Here, indicate whether each material, system or method listed is relevant to your study. If you are not sure if a list item applies to your research, read the appropriate section before selecting a response.

Materials & experimental systems

n/a	Involved in the study	
☐	☒	Antibodies
☒	☐	Eukaryotic cell lines
☒	☐	Palaeontology
☒	☐	Animals and other organisms
☒	☐	Human research participants
☒	☐	Clinical data

Methods

n/a	Involved in the study	
☒	☐	ChIP-seq
☒	☐	Flow cytometry
☒	☐	MRI-based neuroimaging

Antibodies

Antibodies used	Commercial antibodies used: anti-GFP antibody (HT801, Transgen, 1:1000 dilution), anti-plant Actin antibody-HRP (A01050, Abbkine, 1:1000 dilution). Anti-GFP antibody and anti-plant Actin antibody-HRP were detected using Goat Anti-Mouse, HRP conjugated (CW0102S, CWBIO, 1:10000 dilution).<br>Anti-BES1 antibody (1:3000 dilution; kindly provided by J. Li, Lanzhou University, China) was detected using Goat anti-rabbit IgG-HRP (EK020, Zhuangzhibio, 1:5000 dilution).
Validation	Validation statements of commercial primary antibodies are available from manufacturers: anti-GFP antibody (http://www.transgen.com.cn/attached/down/HT801-02_2018010215.pdf), anti-plant Actin antibody-HRP (https://www.abbkine.com/datasheet/A01050.pdf).<br>αnti-BES1 antibody was validated using BL-induced dephosphorylation assays (Fig 1d, 2d, 4e and supplementary Fig 4b).
